# Agro-climatic sensitivity analysis for sustainable crop diversification; the case of Proso millet (*Panicum miliaceum* L.)

**DOI:** 10.1371/journal.pone.0283298

**Published:** 2023-03-23

**Authors:** Eranga M. Wimalasiri, Matthew J. Ashfold, Ebrahim Jahanshiri, Sue Walker, Sayed N. Azam-Ali, Asha S. Karunaratne

**Affiliations:** 1 School of Biosciences, Faculty of Science and Engineering, University of Nottingham Malaysia, Semenyih, Malaysia; 2 Crops for the Future UK, National Institute of Agricultural Botany, Cambridge, United Kingdom; 3 Department of Export Agriculture, Faculty of Agricultural Sciences, Sabaragamuwa University of Sri Lanka, Belihuloya, Sri Lanka; 4 School of Environmental and Geographical Sciences, Faculty of Science and Engineering, University of Nottingham Malaysia, Semenyih, Malaysia; 5 Agricultural Research Council, Pretoria, South Africa; Arab Academy for Science Technology and Maritime Transport, EGYPT

## Abstract

Current agricultural production depends on very limited species grown as monocultures that are highly vulnerable to climate change, presenting a threat to the sustainability of agri-food systems. However, many hundreds of neglected crop species have the potential to cater to the challenges of climate change by means of resilience to adverse climate conditions. Proso millet (*Panicum miliaceum* L.), one of the underutilised minor millets grown as a rainfed subsistence crop, was selected in this study as an exemplary climate-resilient crop. Using a previously calibrated version of the Agricultural Production Systems Simulator (APSIM), the sensitivity of the crop to changes in temperature and precipitation was studied using the protocol of the Coordinated Climate Crop Modelling Project (C3MP). The future (2040–2069) production was simulated using bias-corrected climate data from 20 general circulation models of the Coupled Model Intercomparison Project (CMIP5) under RCP4.5 and 8.5 scenarios. According to the C3MP analysis, we found a 1°C increment of temperature decreased the yield by 5–10% at zero rainfall change. However, Proso millet yields increased by 5% within a restricted climate change space of up to 2°C of warming with increased rainfall. Simulated future climate yields were lower than the simulated yields under the baseline climate of the 1980–2009 period (mean 1707 kg ha^–1^) under both RCP4.5 (–7.3%) and RCP8.5 (–16.6%) though these changes were not significantly (p > 0.05) different from the baseline yields. Proso millet is currently cultivated in limited areas of Sri Lanka, but our yield mapping shows the potential for expansion of the crop to new areas under both current and future climates. The results of the study, indicating minor impacts from projected climate change, reveal that Proso millet is an excellent candidate for low-input farming systems under changing climate. More generally, through this study, a framework that can be used to assess the climate sensitivity of underutilized crops was also developed.

## Introduction

Climate change impacts on regional temperature, rainfall and evaporation patterns can alter meteorological, hydrological, ecological and agricultural interactions [1] and can negatively impact the sustainability of agro-food systems [2]. Global agricultural production is expected to decline as a result of climate change-induced rising temperatures and increased evaporation that lead to water stress and changes in the intensity and distribution of rainfall patterns [3, 4]. It is reported that both yields and calorie content of the world’s major crops, including cassava, maize, rice, sorghum, soybean and wheat, are severely affected by climate change and it is expected that this situation will worsen in the future [5–8].

Various adaptation strategies are developed to avoid yield losses due to climate change [9] and developing resilient agricultural systems is a low-cost and effective strategy to overcome the increased risk of droughts and other climatic hazards [10]. Identification and mainstreaming of crops that are currently neglected or underutilised is central to this process [11]. Underutilised crops that are traditionally grown in low-input systems and are adapted to marginal environments can contribute to the diversification and resilience of agroecosystems, and can potentially contribute to global food and nutrition security. It is therefore important to invest in research and development of evidence-bases for these crops [12–14].

Evaluation of the sensitivity of different crops to changes in climate and to adaptation strategies is a key component in identification and development of measures that have optimum impact. However, most attention is given to simulation of performance for staple/ major crops [10, 15, 16]. While yield forecasting for neglected and underutilised crops is gaining popularity, major work remains to simulate their suitability and response to climate and economic scenarios [7, 17].

Prediction of climate change and the assessment of its impact on crop yield are important to identify adaptation strategies [18]. Crop simulation modelling allows studying the potential of crops and crop varieties in different geographic locations under different climates without the need for extensive agronomic experiments that can be time-consuming and expensive [19]. More specifically, novel crop modelling approaches and supportive databases have been developed to simulate the production of neglected and underutilised crops [20]. In order to overcome the lack of research evidence and to make a rapid progress with the current study, quantification of crop-climate interactions was implemented through the crop modelling approach [10, 16].

Proso millet (*Panicum miliaceum* L.) is an underutilised minor cereal that is a staple food in some parts of Africa and Asia, that is generally found in rainfed farming systems [21]. It is also suitable for cultivation in several geographic locations including Europe [12]. Farmers grow Proso millet as a subsistence crop with local landraces for dietary requirements as well as for income generation [22]. The primary sources of demand for this crop are the rural farming population where people prefer it as a substitute for rice [23]. It is rich in protein content (12.5%) and contains higher nutritive (vitamins, minerals, essential amino acids, fats and dietary fiber) values compared to major cereals such as rice, maize and wheat [21, 24, 25]. The crop has a short growing period of around 70 days and requires a low amount of water to develop and yield [21, 26]. The available literature suggests that Proso millet is a stress-tolerant plant and produces reasonable yields where other crops like maize and paddy fail to grow or give poor yields [27, 28]. Proso millet is cultivated under harsh environmental conditions in Sri Lanka where other crops (paddy, vegetables, maize and groundnuts) fail in the dry season [29]. It is also a good candidate in crop rotations to control weeds, disease and pest cycles [30]. The ability of Proso millet to grow in a wide range of soils and climates suggests suitability as a future crop under climate change [31]. However, the climate sensitivity, and yield projections for Proso millet under climate change, are not well documented. However, growth data from regions in which proso millet is cultivated can be used to determine the degree of resiliency of this crop against a wide range of current and future climates.

With the substantial uncertainty in climate change projections [32], it is important to study the resilience of drought-tolerant underutilised minor millets such as Proso millet. Therefore, this paper aims (i) to evaluate the sensitivity of Proso millet production to temperature and precipitation variability, (ii) to analyse the projected future climate (2040–2069) of a known Proso millet growing area using the data from 20 general circulation models (GCMs) under different emission scenarios, (iii) to project the crop yields under future climate scenarios in the growing region and (iv) to identify the potential for Proso millet production areas across Sri Lanka under climate change. This paper describes the first study that estimates the potential production of Proso millet using a crop modelling approach which also can be applied to other underutilised crops (see discussion section).

## Materials and methods

### Location details

As trial data for modelling is scarce for Proso millet, data from a case study in Sri Lanka was chosen to evaluate the agro-climatic sensitivity of this crop. Sri Lanka is a small tropical island in the Indian ocean with year-round warm weather. The country is vulnerable to climate change in terms of rainfall variability and an increase in climate extremes and warming [33]. Most farmers and consumers in the country are expected to be adversely affected by climate change, and cultivation of several crops including the staple rice will be at risk due to limitation of water [15].

The suitability of five Proso millet samples (hereafter named as accessions), which were named as L_1, L_11, L_12, L_14 and L_25 [23], was evaluated for a known Proso millet growing region (6.428°N, 81.090°E) in Bodagama, Sri Lanka. These 5 accessions were not cultivated in all locations, with sampled fields each typically containing a single accession. This area belongs to the Low County Dry Zone region (DL1b) of Sri Lanka, which receives 1100–1750 mm of annual rainfall. The Northeastern monsoon that falls during December–February is the predominant rainy season, while Proso millet is cultivated during a secondary rainy season in March–June. The rainfall characteristics of the study area were previously described in Wimalasiri et al [29].

### Climate data

#### Baseline (1980–2009) climate data

A complete observed climatic dataset for the 1980–2009 period was not available in the cultivation area. Thus, as observed climate data, we used daily rainfall observations at Thanamalwila (6.44°N, 81.13°E, for 1989–2009), the closest (4.6 km away from the cultivation location) meteorological station to the growing area along with daily minimum and maximum temperatures at Sewanagala (6.40°N, 80.91°E, 1986–2009, 20.1 km from the cultivation location). Gaps in these observed datasets were filled by NASA Modern Era Retrospective-analysis for Research and Applications (MERRA) [34] data. The MERRA data were previously used in Sri Lanka for gap filling in climate change studies [15]. Neither meteorological station recorded solar radiation, therefore MERRA data were used.

#### Mid-21^st^ century (2040–2069) climate data

The climate and yield projections include 30 growing seasons for mid-21^st^ century (2040–2069), using downscaled climate model data. The climate change scenarios were obtained from 20 GCMs (labelled A–T in S1 Table) in the Coupled Model Intercomparison Project (CMIP5) archive [35], which were selected as those available for both RCP4.5 and RCP8.5 in the online data portal (http://ccafs-climate.org/) of Consultative Group for International Agricultural Research (CGIAR) Research Program on Climate Change, Agriculture and Food Security (CCAFS).

Two Representative Concentration Pathways (RCPs) were selected for this study as; RCP4.5 represents a medium-lower concentration scenario that is broadly consistent with current global pledges for mitigation policies, and RCP8.5 represents an extreme high emission scenario [36]. Daily rainfall, minimum and maximum temperatures and solar radiation data for mid-century (2040–2069) growing seasons were downscaled based on the delta method [37]. The delta method assumes that the relationships between variables of the current (baseline) climate are maintained towards the future and changes in climate are relevant at coarse scales [38]. Therefore, the GCM in the following sections indicates downscaled future climate data using the delta method.

### Crop model

The calibrated Agricultural Production Systems Simulator (APSIM) [39] millet model (version 7.8) was used to simulate the effect of different realizations (Section 2.4) of climate variability and climate change on the yield of the five Proso millet accessions (L_1, L_11, L_12, L_14, L_25). A crop module for Proso millet is not available in the APSIM model, therefore, the millet model was used. The model description, calibration and validation procedures were described in detail [23], therefore a brief summary is presented here.

#### Model description

Daily growth and development of Pearl millet crop was simulated in APSIM millet model. The model was developed based on the field experimental data from ICRISAT–Patancheru, India [40–42]. In phenology of millet module, there are 11 crop growth stages and thus, 10 growth phases; sowing, germination, emergence, end of juvenile, floral initiation, flag leaf, flowering, start grain fill, end of grain filling, maturity and harvest ripe. Soil moisture controls sowing to germination stages while the accumulation of thermal time determines all other growth stages. The daily thermal time is decreased by nitrogen or water stress between emergence and flag leaf stages, which delays phenology. Daily biomass accumulation is a function of soil water (for transpiration) and radiant energy. Accumulation of thermal time or biomass determines the rate of tiller appearance. Once the crop is harvested, residues pass to other modules (residue2 and soiln2). The base, optimum and maximum temperatures used in the model were 10°C, 30°C and 45°C respectively. The genotype coefficients of 5 calibrated Proso millet accessions are mentioned in Table 1 [23].

**Table 1:** Accession specific genotype coefficients used to calibrate APSIM model.

Parameter	Unit	L_1	L_11	L_12	L_14	L_25
**Potential grains per head**	grains/head	2940	2645	3140	2795	3210
**Potential grain growth rate**	mg/grain/d	0.61	0.61	0.61	0.61	0.61
**TT from emergence to end of juvenile phase**	°C days	348.5	322	345	331	339
**Photoperiod sensitivity**	°Cd/h	112.4	112.4	112.4	112.4	112.4
**TT from flowering to maturity**	°C days	440	466	437	450	457
**TT from flag leaf to flowering**	°C days	85	90.5	94	87	70
**TT from flowering to start grain fill**	°C days	83	80	86	91	95
**TT from maturity to harvest ripe**	°C days	1	1	1	1	1

### Yield simulations

Proso millet yield under different climate scenarios (Table 2) was simulated using the above calibrated model. The period from 15^th^ March to 15^th^ June was considered the Proso millet growing season. Crop management practices, soil and climate data used in APSIM simulations are previously described [23]. It was assumed that crop management practices such as planting and fertiliser application dates and amount of fertiliser are similar in all the simulations, with the only difference in the climate data.

**Table 2:** Different types of yield simulations performed using the APSIM model.

Input to APSIM simulation	Description
**Baseline climate**	Climate data for 1980–2009 based on weather station observations with MERRA gap filling for the Proso millet growing area in Bodagama, Sri Lanka.
	(30 years x 5 accessions)–Section Baseline (1980–2009)–Section: Climate Data
**CMIP5 climate change**	Baseline climate data with temperature and precipitation anomalies (‘Delta’ for 2040–2069 vs 1980–2009) from CMIP5 models under RCP4.5 and RCP8.5
	(30 years x 5 accessions x 20 models x 2 scenarios)–Section: Mid-21^st^ Century (2040–2069) Climate Data
**C3MP climate change**	Baseline climate data with 99 combinations of temperature and precipitation anomalies within C3MP-defined climate change space
	(30 years x 5 accessions x 99 anomaly combinations)–Section: Coordinated Climate-Crop Modelling Project
**Sri Lanka baseline**	Climate data for 1980–2009 from MERRA for a 0.25° grid of 95 locations covering Sri Lanka
	(30 years x 5 accessions x 95 locations)–Section: Mapping of yield potential and sensitivity
**Sri Lanka climate change**	Sri Lanka baseline climate data with 5 idealized temperature or precipitation anomalies
	(30 years x 5 accessions x 95 locations x 5 anomalies)–Section: Mapping of yield potential and sensitivity

### Coordinated Climate-Crop Modelling Project (C3MP)

The Agricultural Model Intercomparison and Improvement Project (AgMIP) developed Coordinated Climate-Crop Modelling Project (C3MP) to study the sensitivity of crop yields to changes in [CO_2_], temperature and rainfall by 99 combinations of these variables [10, 16]. In contrast to climate projections using GCMs, C3MP analysis is not based on emission scenarios, is not climate or location dependent, but instead is universal to, and comparable across, every agricultural land in the world [10]. The C3MP sensitivity tests are designed to test the combinations of temperature, rainfall and carbon dioxide changes at the end of 21^st^ century, within defined ranges (Table 3). The C3MP has extended the climate metric ranges slightly beyond the climate extremes projected by GCMs in CMIP5 [16].

**Table 3:** Climate metric ranges for C3MP climate sensitivity experiments.

Climate Metric	Lower Bound	Upper Bound
**Temperature change**	–1°C	+8°C
**Rainfall change**	–50%	+50%
**Carbon dioxide concentration**	330 ppm	900 ppm

This range covers the majority of cropping areas in the world, although larger percentage changes to small baseline rainfall amount are expected in some arid regions [10]. The 99 climate sensitivity tests, generated using the Latin Hypercube sampling method [10], were applied to the baseline (1980–2009) daily weather data.

The APSIM millet model is not sensitive to CO_2_ as it was not parameterised for variations in CO_2_ concentration. Being a C4 plant, the response of Proso millet to CO_2_ variations will be smaller than for C_3_ plants, therefore it was not expected that the reduction of yield due to increased temperature will be offset by increased CO_2_ [43]. Hence, CO_2_ variation was omitted, and the analysis proceeded with varying rainfall and temperature only. The issues and possible outcomes of not including CO_2_ variations are covered in the discussion section. The climate control option of the APSIM model was used to create the 99 climate sensitivity simulations.

### Mapping of yield potential and sensitivity

Ninety-five locations in 0.25° resolution grids were selected across Sri Lanka for mapping (S1 Fig). Daily meteorological data for all the locations during 1980–2009 period were collected from Nasa MERRA. Site specific soil data were obtained from Soilgrids [44]. Five hypothetical climate change scenarios were prepared as +1°C, +1.5°C and +2°C temperature increments and +25% and -25% rainfall changes, relative to the baseline climate. All simulations were performed using the calibrated APSIM millet model. It was assumed that sowing date (which is in the minor growing season) and crop management practices are the same in all 95 locations. Spatial interpolations between the 95 locations were done according to the Inverse Distance Weighting (IDW) model using ArcMap 10.7.1.

## Results

### Proso millet simulation for baseline (1980–2009) period

The average yield for the baseline climate (1980–2009) period ranged from 1622 kg ha^–1^ (SD 941 kg ha^–1^) for L_14 to 1823 kg ha^–1^ (SD 1036 kg ha^–1^) for L_25 with a mean of 1707 kg ha^–1^ for all accessions. The simulated yields of five different accessions (for whole 30 years) were not significantly different (p > 0.05) form each other. Out of 30 years, 15 years (50%) showed higher yields than the average (1707 kg ha^–1^) while the rest of the years were below average. The reported lower limit of seasonal rainfall above which Proso millet gives a satisfactory yield is 300 mm [45]. However, out of all the growing seasons in the baseline period, the rainfall in 57% of seasons were below this limit.

**Fig 1:**
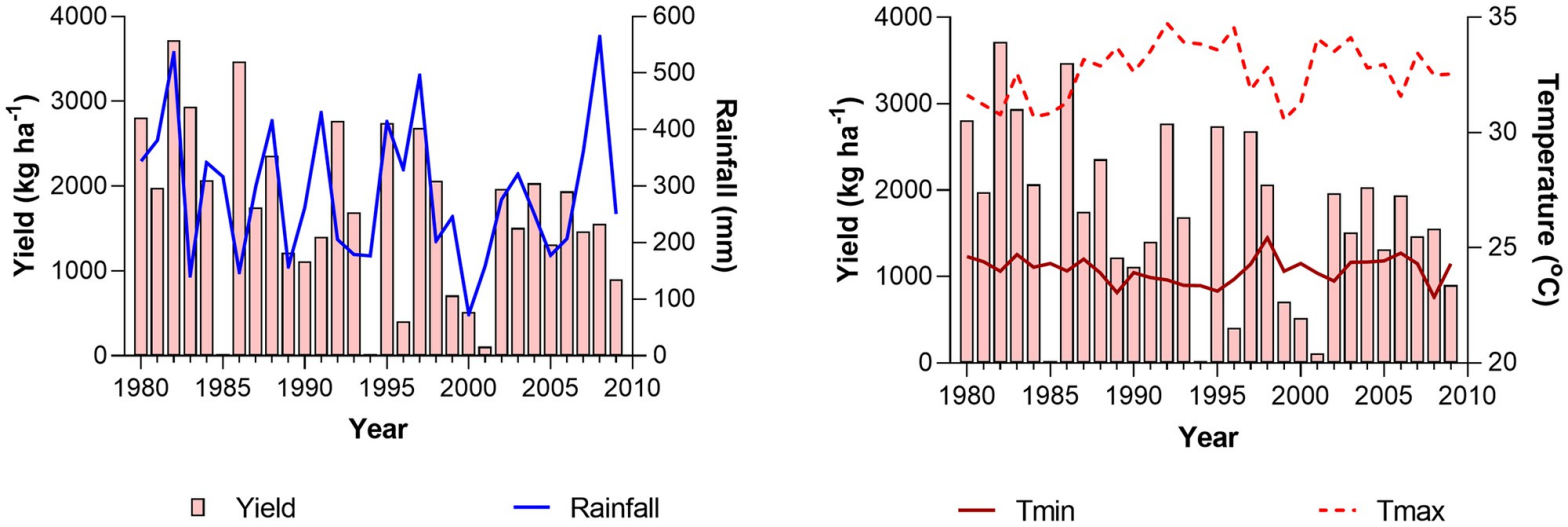
Variation of average simulated Proso millet yield of five accessions and climate parameters during Proso millet growing season in the baseline (1980–2009) period. The Tmin and Tmax stand for seasonally averaged minimum and maximum temperatures respectively.

The average yield variation of five accessions showed a negative trend (42.5 kg ha^–1^ per year) throughout the 1980–2009 period and it was significantly different from zero (p = 0.0384) at 95% confidence level. The variation of average Proso millet yield of five accessions and seasonal climate parameters (rainfall, minimum and maximum temperatures) during the growing season is shown in Fig 1.

### Proso millet production sensitivity according to the C3MP analysis

**Fig 2:**
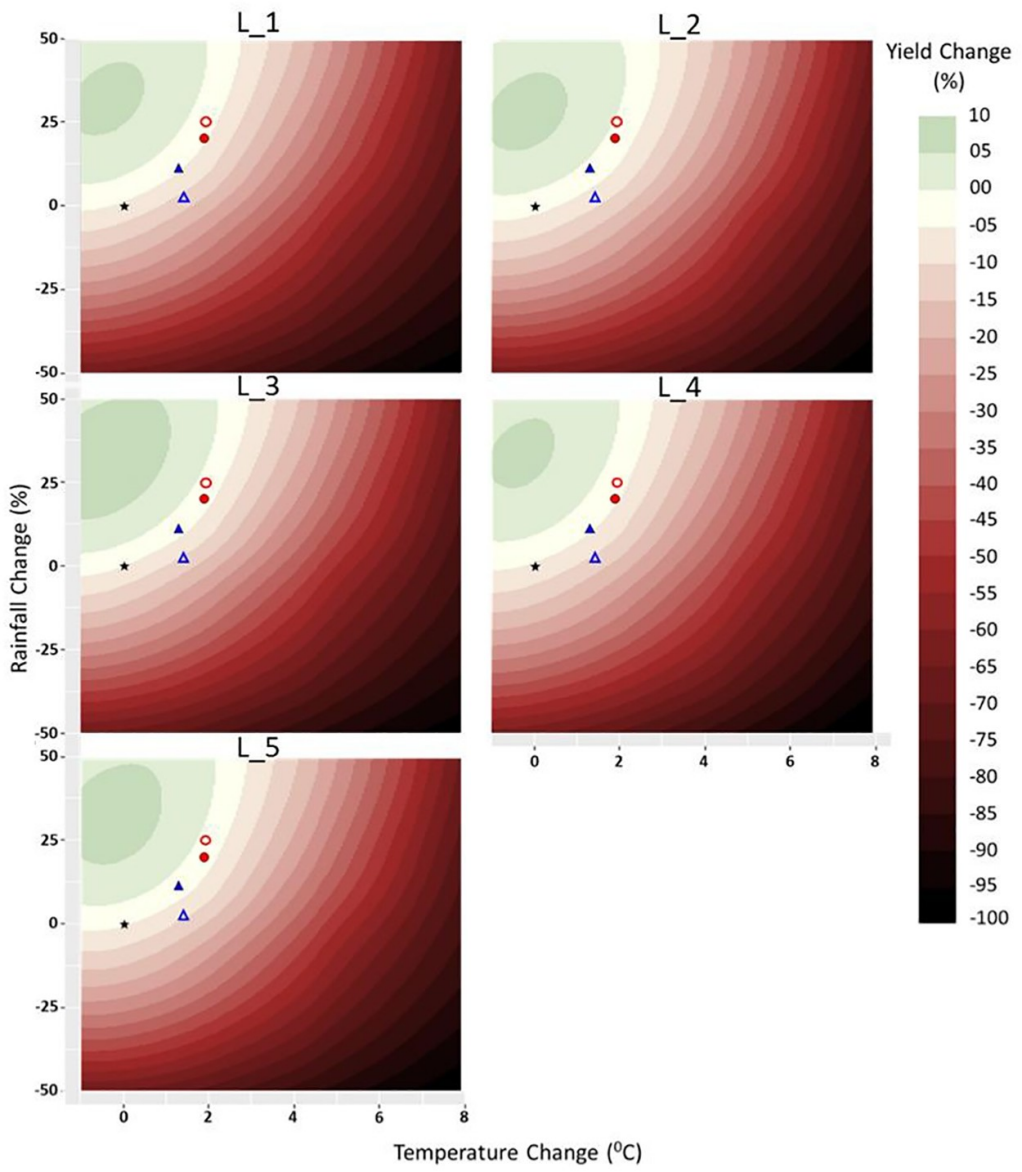
Yield sensitivity of five Proso millet accessions to rainfall and temperature changes in Dry Zone, Sri Lanka under C3MP and mid-century climate, where filled blue triangle is mean annual rainfall (P) and temperature (T) changes for RCP4.5, red dot is annual P and T change for RCP8.5, open triangle is mean Proso millet growing seasonal P change, open dot is mean Proso millet growing seasonal T change and star is yield sensitivity at zero P and T change.

For all accessions simulated Proso millet yield increased by around 5% within a restricted climate change space of up to 2°C of warming and with increased rainfall (Fig 2). Accession L_12 showed the highest yield increment (10%) at 1°C of warming under wetter conditions (50% increment of rainfall). The yield decreased with increasing temperature in all the tested accessions. For example, yield reductions at 1°C, 2°C and 3°C increments with unchanged rainfall were 5–10%, 10–20%, and 20–30% respectively. Temperature reduction of –1°C without changing rainfall did not reduce Proso millet yield for all the accessions, suggesting an ability to withstand slightly cooler environments.

Proso millet yield also decreased with the reduction of rainfall in all the tested accessions. A 25% and 50% reduction of rainfall at zero temperature change reduced the yield by 25–30% and 60–65% respectively. Proso millet sensitivity to rainfall increment showed a similar pattern in all accessions but in different magnitudes. However, the highest rainfall increment did not lead to the highest yield increment (Fig 2). The accession L_12 gave the highest yield in a wide range of rainfall increments (14–46%) suggesting its ability to withstand wetter conditions. The pattern of increased yields with a medium rainfall increment (25%) and decreased yields with a higher rainfall increment (+50%) at zero temperature change is consistent across accessions.

### Mid-21^st^ century climate of Proso millet growing area

The annual and Proso millet growing seasonal (15^th^ March to 15^th^ June) climate during the observed baseline period (1980–2009) and over the mid-21^st^ century (2040–2069) for the downscaled 20 GCMs under RCP4.5 and RCP8.5 are summarised in Table 4.

**Table 4:** The annual mean maximum and minimum temperatures, annual and growing seasonal (15th March– 15th June) rainfall for the baseline period (1980−2009) and in mid of the 21st century (2040−2069) for 20 general circulation models (downscaled using delta method) under the RCP4.5 and RCP8.5 scenarios in Bodagama, Sri Lanka.

Model	RCP4.5				RCP8.5			
	Mean Tmax (°C)	Mean Tmin (°C)	Total annual rainfall (mm)	Seasonal rainfall (mm)	Mean Tmax (°C)	Mean Tmin (°C)	Total annual rainfall (mm)	Seasonal rainfall (mm)
**Baseline**	32.1	23.3	1121	289				
**BCC-CSM1-1 (A)**	33.1	24.4	1124	321	33.4	24.7	1360	338
**BCC_CSM1_1_M (B)**	33.2	24.3	1065	249	33.5	24.7	1107	253
**BNU-ESM (C)**	33.2	24.5	1249	298	33.7	25.0	1331	344
**CanESM2 (D)**	33.7	24.8	1264	300	34.5	25.6	1661	445
**CSIRO-Mk3-6-0 (E)**	33.3	25.0	1628	222	34.2	25.4	1291	313
**GFDL_CM3 (F)**	34.2	25.3	1300	333	34.8	26.1	1908	1088
**GFDL-ESM2G (G)**	33.2	24.3	1247	445	33.7	25.0	1050	257
**GFDL-ESM2M (H)**	33.4	24.4	1045	228	33.8	25.0	995	198
**INMCM4.0 (I)**	32.6	23.8	1084	282	33.0	24.3	1046	285
**IPSL-CM5A-LR (J)**	33.7	24.9	1383	308	34.6	25.5	935	169
**IPSL-CM5A-MR (K)**	33.5	24.9	1331	319	34.3	25.5	1800	279
**MIROC-ESM (L)**	33.2	24.7	1244	274	34.0	25.2	1265	305
**MIROC_ESM_CHEM (M)**	33.0	24.7	1226	303	34.2	25.4	1346	355
**MIROC5 (N)**	33.3	24.5	1225	296	33.9	25.1	1896	491
**HadGEM2-CC (O)**	33.4	24.8	1169	290	34.1	25.3	1284	358
**HadGEM2-ES (P)**	33.6	25.0	1222	303	34.2	25.4	1200	377
**MPI-ESM-LR (Q)**	33.4	24.6	1239	254	34.1	25.2	1129	268
**MPI-ESM-MR (R)**	33.3	24.7	1438	334	34.1	25.2	1160	287
**MRI-CGCM3 (S)**	33.1	24.3	1213	275	33.6	24.7	1235	462
**NorESM1-M (T)**	33.1	24.3	1227	314	33.7	24.9	1798	349
**Multi-model mean**	33.3	24.6	1246	297	34.0	25.2	1340	361

The mean maximum temperature (Tmax) and minimum temperature (Tmin) of the observed baseline period (1980–2009) were 32.1°C and 23.3°C respectively. The mean Tmax and Tmin increased in all the GCMs under both RCP4.5 and RCP8.5 scenarios. The mean Tmax increased by 0.6°C (INM_CM4) to 2.1°C (GFDL_CM3) in RCP4.5 and by 1.0°C (INMCM4.0) to 2.8°C (GFDL_CM3) in RCP8.5. The mean Tmax for 2040–2069 period under RCP4.5 and RCP8.5 was 33.3°C and 34.0°C respectively.

The annual and Proso millet growing seasonal rainfall during the observed baseline period was 1121 mm and 289 mm respectively. Under RCP4.5, seventeen GCMs (85%) showed an increment of annual rainfall while 3 GCMs (15%) exhibited a decrease in annual rainfall compared to the baseline period. The annual rainfall change ranged from -6.8% (GFDL_ESM2M) to +45.2% (CSIRO_Mk3_6_0) under RCP4.5. Proso millet growing seasonal rainfall increased in 13 GCMs (65%) and decreased in 7 GCMs (35%) for the RCP4.5 emission scenario. Proso millet growing seasonal rainfall change ranged from -23.2% (CSIRO_Mk3_6_0) to +54% (GFDL_ESM2G).

Under RCP8.5, the annual rainfall increased in 15 GCMs (ranged from +0.8% in MPI-ESM-LR to +70.3% in GFDL_CM3). The IPSL-CM5A-LR recorded the highest annual rainfall reduction (-16.6%) for RCP8.5. Rainfall during Proso millet growing season increased in 12 GCMs (60%), with 8 GCMs (40%) projecting a rainfall reduction.

GFDL_CM3 was identified as an outlier under the RCP8.5 scenario for Proso millet growing seasonal rainfall, projecting an average of 1088 mm (+277% compared to the baseline) for 2040–2069, compared with an average of 323 mm from the 19 other GCMs for the same period. However, annual and Proso millet growing seasonal rainfall of GFDL_CM3 under RCP4.5 did not show extreme values (Table 4).

However, 50% of GCMs under RCP4.5 and 40% of GCMs under RCP8.5 were below the lower limit of seasonal rainfall above which Proso millet gives a satisfactory yield [45]. Comparatively higher variation was recorded for both annual (CV–coefficient of variation 0.11) and Proso millet growing seasonal (CV 0.16) rainfall under RCP4.5 than the RCP8.5 scenario where the CV values were 0.23 and 0.52 in respective season under RCP8.5.

### APSIM millet yield prediction for the mid-21^st^ century

The predicted Proso millet yields from the APSIM millet model, using downscaled climate data for mid-century (2040–2069) under RCP4.5 and RCP8.5 scenarios were statistically analyzed for deviations from the baseline (1980–2009) yields. It was found that Proso millet yields of all the tested accessions (L_1, L_11, L_12, L_14 and L_25) were not significantly (p > 0.05) different from the baseline yields under both RCP4.5 and RCP8.5 scenarios. The average yield of all the accessions in all GCMs under RCP4.5 and RCP8.5 were 1584 kg ha^–1^ and 1425 kg ha^–1^ respectively.

Taking the average across all GCMs, mean Proso millet yields of all the accessions simulated by APSIM were lower than the baseline (1980–2009) yield under both RCP4.5 (7.3% reduction) and RCP8.5 (16.6% reduction) scenarios for 2040–2069 period. These values are close to the yield change values expected based on the C3MP analysis (0–10% reduction) when only the rainfall and temperature sensitivity on Proso millet yield were analysed. However, it should be noted that there is no change in the rainy days in the C3MP analysis as the modifications (precipitation in %) were applied to the baseline climate.

**Fig 3:**
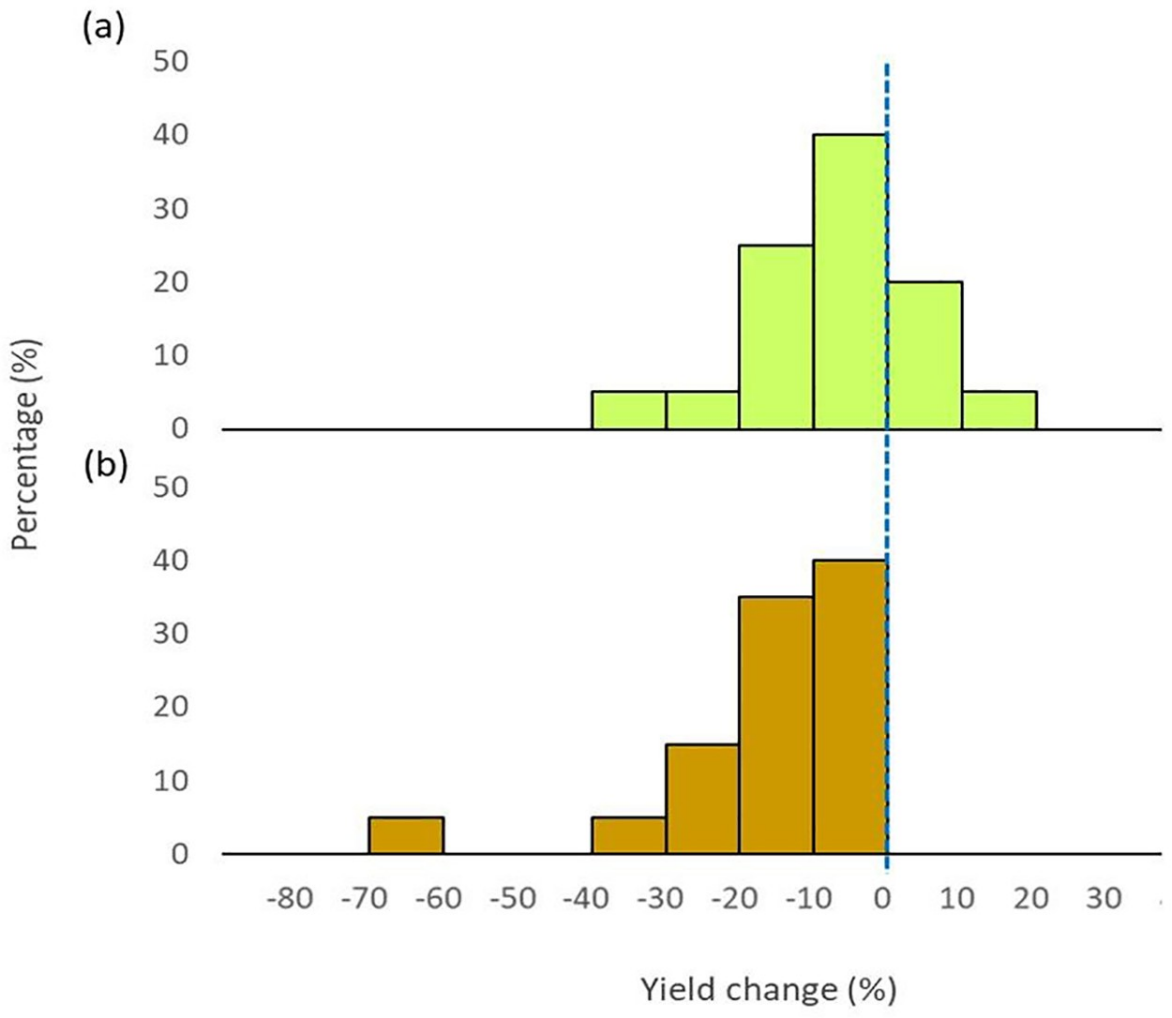
Relative Proso millet yield change (average of 5 accessions) for the 2040–2069 period with compared to baseline (1980–2009) period in all GCMs under RCP4.5 (a) and RCP8.5 (b).

The variation of future climate among different models led to the variation of Proso millet yield in the mid-21st century. In RCP4.5, the average yield of 5 Proso millet accessions increased in 25% of GCMs (Fig 3). Among the yield change bins in Fig 3, the highest percentage of climate models (40%) simulated climates that led to a decrease in Proso millet yield by 0–10% below the average for the baseline period. Simulated climate changes led to a Proso millet yield reduction greater than 20% in 10% of GCMs. In RCP8.5, the yield decreased by 0–10% in 40% of GCMs followed by 10–20% in 35% of GCMs. Only 5% of GCMs (one model) recorded a yield reduction greater than 60% from the baseline. None of the GCMs showed a yield increment under the RCP8.5 scenario.

Out of 20 GCMs, only four (BCC-CSM1-1, GFDL-ESM2G, IPSL-CM5A-MR and MIROC_ESM_CHEM) showed yield increment for 2040–2069 period under RCP4.5 scenario in all the accessions (S2 Fig). The MPI-ESM-MR showed yield increment in all four accessions except L_14.

### Proso millet yield potential map

**Fig 4:**
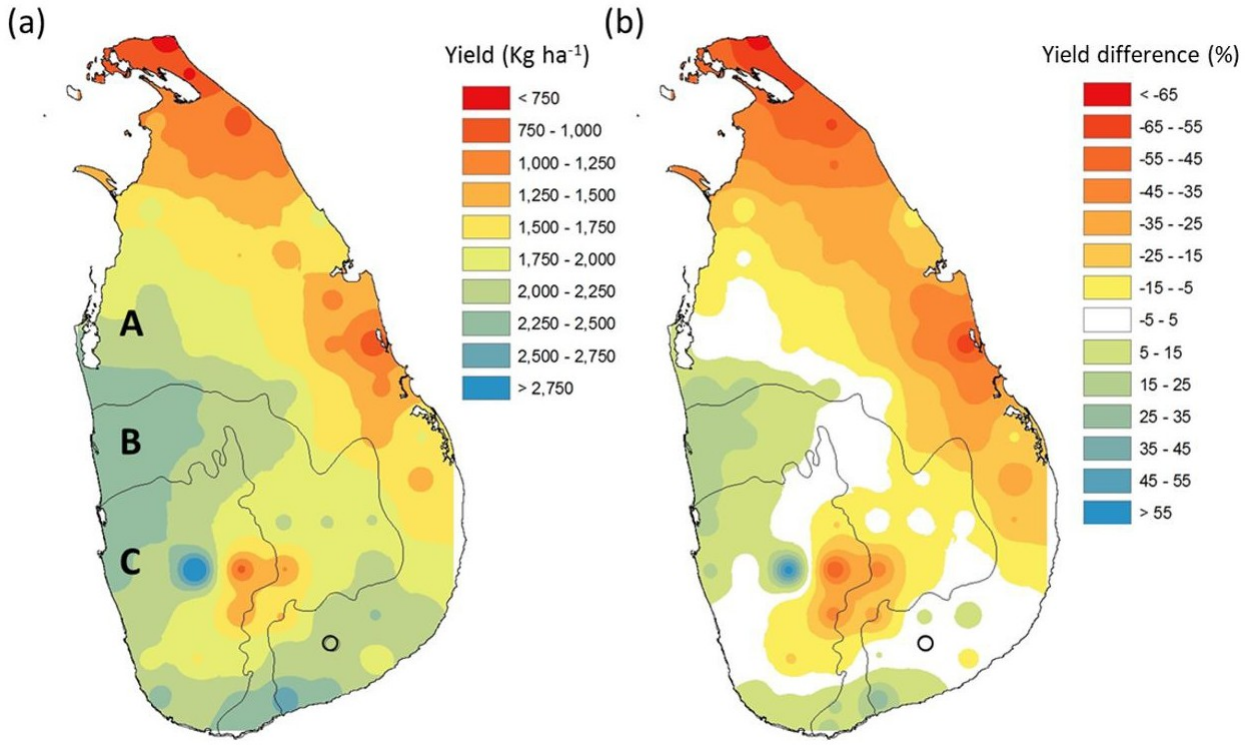
(a) Potential average Proso millet yield of five accessions and (b) yield difference compared to the currently growing area (circled) during 1980–2009 period (the boundary was obtained from https://data.humdata.org/dataset/cod-ab-lka).

The gridded MERRA data was used as an input to APSIM simulations to cover all of Sri Lanka. No significant (p > 0.05) difference was reported between the simulated yield from the gridded data and observed data (gap-filled) at the experimental site in Thanamalwila, Sri Lanka for the baseline (1980–2009) period (average yield 1707±984 kg ha^-1^). The potential Proso millet yield and the yield difference compared to the experimental site is shown in Fig 4.

The average yield for the baseline period varied from 617 kg ha^-1^ to 3282 kg ha^-1^ with an average of 1818± 491 kg ha^-1^ across the country. When compared to the study site, simulated Proso millet yield is different by between -70.1% to +59.1% across the country. Comparatively lower yields were reported for the North and Eastern parts of the country where North-East Monsoon is the prominent rainy season (Fig 4a). The yields are also lower in central highlands which is currently used for tea cultivation due to cooler climate. An unusual higher yield was reported from one location in the Wet Zone (Fig 4).

Out of the all locations, 31.9% showed higher yields than the study area while 21.3% belonged to -5 to +5% yield change category. The yield is higher in parts of the Intermediate and Wet Zones, where Proso millet is not normally cultivated at present. The southern part of the Dry Zone also showed a yield increment when compared to the current growing area. This indicates a huge potential exists for Proso millet cultivation in other locations of the country during the minor growing season (15^th^ March– 15^th^ June). All the maps can be downloaded from https://doi.org/10.5281/zenodo.7456224.

**Fig 5:**
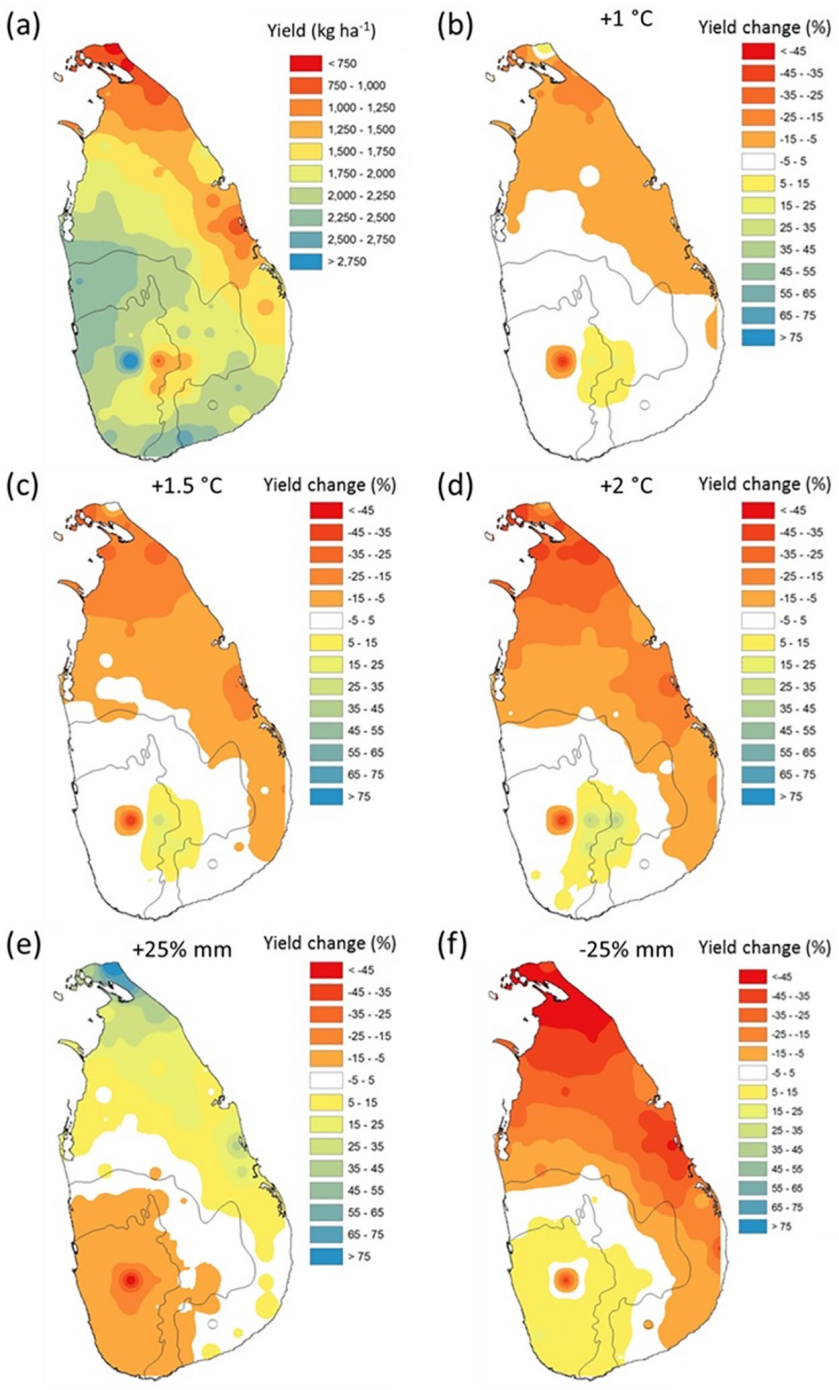
(a) Average yield of Proso millet accession L_12 for 1980–2009 period and yield change under (b) 1°C, (c) 1.5°C and (d) 2°C temperature increment, (e) 25% rainfall increment and (f) 25% rainfall reduction compared to the baseline (1980–2009) climate (the boundary was obtained from https://data.humdata.org/dataset/cod-ab-lka).

The impact of climate change on hypothetical Proso millet yields across Sri Lanka was studied using accession L_12, which showed the closest yield to the average baseline yield (Fig 5). The yield of accession L_12 was not significantly (p > 0.05) different from the average yield of five accessions. Uniform increments of temperature by 1°C, 1.5°C and 2°C increased the yield in 30.9%, 33.0% and 25.5% of locations respectively.

Rainfall increment by 25% increased the average yield (1837±367 kg ha^-1^) while 25% reduction of rainfall decreased the average yield (1630±685 kg ha^-1^). A higher yield variability was observed under the rainfall reduction scenario. Yield under climate change clearly showed that yields behave in different ways in different climatic zones. The increased yield and lower reduction under increased temperature and decreased rainfall showed the tolerability of Proso millet to harsh agroclimatic conditions and suggest the possibility of use of the crop under climate change.

## Discussion

The future temperature projections recorded in this study are consistent with the Intergovernmental Panel on Climate Change (IPCC) global projections [46]. The IPCC [46] projected an increment of global temperature by 1.4°C (ranged from 0.9°C to 2°C for RCP4.5) and 2.0°C (ranged from 1.4°C to 2.6°C) for RCP8.5 during 2046–2065 period (IPCC 2013). The mean increment of temperature in Proso millet growing region in Sri Lanka for the 2040–2069 period (1.3°C and 1.9°C for RCP4.5 and RCP8.5 respectively) are in alignment with the IPCC [46] projected range. Further, the projected temperature increment in Proso millet growing region agrees with the previous studies (1.5–2.8°C for minor and 1.1–2.4°C for major season) in North Western province of Intermediate Zone of Sri Lanka for 2040–2070 period [15] and Walawe basin in the Southern part of the country [47]. However, rainfall projections showed both higher and lower values for mid-century than the baseline period that stems from the uncertainties among climate models. A higher model uncertainty was reported in the precipitation projections over Sri Lanka for 1901–2100 period when compared with India [48] and the present study confirms these findings. The small spatial scale considered in the study [15, 32] hindered the accurate comparison and discussion on model uncertainty and variability. This should be better answered by more data from different geographic locations.

The projected future climate of 20 GCMs showed variation among models. Out of 20 GCMs used in this study, the GFDL_CM3 projected an extreme rainfall for April (RCP8.5) while it was not an outlier under RCP4.5 (Table 4). The GFDL_CM3 is one of the best performing models to predict Indian Summer Monsoon (June–September) in different timescales (up to year 2099 –[49]; year 2051 to 2099 period–[50]) under the RCP8.5 scenario. The predicted extreme rainfall belongs to First Inter Monsoon while the studies were done for South West Monsoon period in Sri Lanka (Indian Summer Monsoon). However, the rainfall during June–September period was predicted well by the GFDL_CM3. The implausible increment of April rainfall to 900 mm from this model cannot be addressed by the available literature, therefore, further studies are needed on the behaviour and strength of GFDL_CM3 over Sri Lanka, specially under the First Inter Monsoon period. The multimodal mean with and without GFDL_CM3 were studied, therefore it will not affect the results of the current study.

Proso millet yields in all the tested accessions are likely to decrease with the expected warming, regardless of the variation of rainfall, however, low rainfall makes this more severe. According to Sharmila et al. [50] there are three key processes that cause yield reduction of millets and sorghum in warmer environments: i) increased potential evapotranspiration in water scarce soils limits the water availability for roots to uptake, ii) increased maintenance respiration per biomass unit decreases biomass production and iii) reduced length of crop cycle also lowers the biomass production.

According to the model output, the sensitivity of Proso millet to rainfall which was generated using C3MP approach showed that the highest yield was not from the highest rainfall increment. It was reported that heavy rains after two weeks of planting Proso millet are destructive, causing poor plant stand by covering seeds too deep to emerge or create soil surface crust difficult for seedlings to penetrate [51]. Nielsen and Vigil [52] studied the effect of environmental parameters influence on Proso millet yield and found that the rainfall during 12–18 August (59–65 DAS) has the highest impact on yield, where sowing was 14 June and harvesting was 15 September. Further studies are needed on the most effective and destructive rainfalls during Proso millet lifespan.

Different GCMs, time slices, crops, locations and methodologies hamper proper comparison of the yield projections of this study with other studies. However, based on the similar approach, but using APSIM and DSSAT models driven by future climate that has 4 GCMs (RCP8.5) similar to the present study (GFDL-ESM2M, MIROC5, HadGEM2-ES and MPI-ESM-MR), Zubair et al. [15] projected a reduction of Paddy yield by 28%, 33%, 41% and 36% respectively in mino*r* season in the Intermediate Zone of Sri Lanka. In contrast, APSIM simulations reported by Zubair et al. [15] for the same season in the same location recorded yield increment in three GCMs (ranged from 3–9%) except HadGEM2-ES where a 2% reduction was observed. Proso millet yield reduction in this study are smaller than DSSAT simulations (39%, 9%, 13% and 5% different cultivars respectively), but larger than the APSIM simulations. It should be noted that paddy farming includes extensive use of irrigated water, fertiliser and crop husbandry practices that are not presently used in Proso millet cultivation. Further, the agroecological zone and soil characteristics of the study area are also different for the present study.

One of the limitations of the present study is that the APSIM millet model was not calibrated for CO_2_, therefore the effect of CO_2_ on Proso millet yield could not be studied. Being a C_4_ plant, it is not expected that a higher yield reduction due to increased temperature is counteracted by the increased atmospheric CO_2_, because the response of C_4_ plants to increasing CO_2_ is lesser than the C_3_ plants [43]. The carbon dioxide concentration is 3 to 6 times higher within C_4_ plants than the atmospheric CO_2_ concentration because RuBisCO (ribulose-1,5-biphosphate carboxylase-oxygenase), that reacts with CO_2_ are localised to bundle sheath cells [43]. Therefore, the cells saturate, and RuBisCO prevents the increasing CO_2_ uptake with increasing CO_2_ concentration [53]. However, reduction of stomatal conductance that increases the water use efficiency may increase the yield in C_4_ plants [54]. It was found that doubling the carbon dioxide approximately increased yield by 30% in C_3_ species and less than 10% in C_4_ species [55]. However, previous studies showed that the increasing CO_2_ caused yield increment in sorghum [56], a C_4_ plant that is also cultivated in Sri Lanka. Under drought conditions, a beneficial effect from enriched CO_2_ is prominent in C_4_ crops like maize due to higher radiation use efficiency and water use efficiency [57]. Therefore, Proso millet that is cultivated under moisture stress conditions can take advantage from the increased CO_2_. Further studies are needed on the CO_2_ sensitivity of Proso millet. However, it is necessary to use a different crop modelling tool to study the effect of changes in CO_2_ on Proso millet yield since current version of the APSIM millet does not represent CO_2_ changes.

The severity of climate change impact on crop yield depends on the crop variety and the region of cultivation [58]. It was found that different crop models play in different ways in the same location [15], therefore this should also be evaluated. None of the climate projections lead to predictions within APSIM of the maximum potential Proso millet yield (4 t ha^–1^) in the mid-21^st^ century in the growing region in the country. In Sri Lanka, Proso millet is found in subsistence farming systems with very low inputs such as no artificial irrigation and fertiliser application that was observed rarely [29]. Therefore, it is important to study the soil moisture conservation, water management, fertiliser application and advanced crop husbandry technologies and their impact on future yield and climate sensitivity of Proso millet in Low Country Dry Zone Sri Lanka.

The climate beyond the mid-21^st^ century (2040–2069) was not studied in this work. It is projected that global mean surface temperature will be increased by 1.8°C (ranged from 1.1°C to 2.6°C) under RCP4.5 and 3.7°C (ranged from 2.6°C to 4.8°C) under RCP8.5 scenario by the end of the century (2081–2100) relative to the 1986–2005 period [46]. Further, there are larger differences between the RCP4.5 and RCP8.5 scenarios at the end of the century than during mid-century [46]. Therefore, it is expected that yield losses in the end-21^st^ century would be much larger under the RCP8.5 scenario.

**Fig 6:**
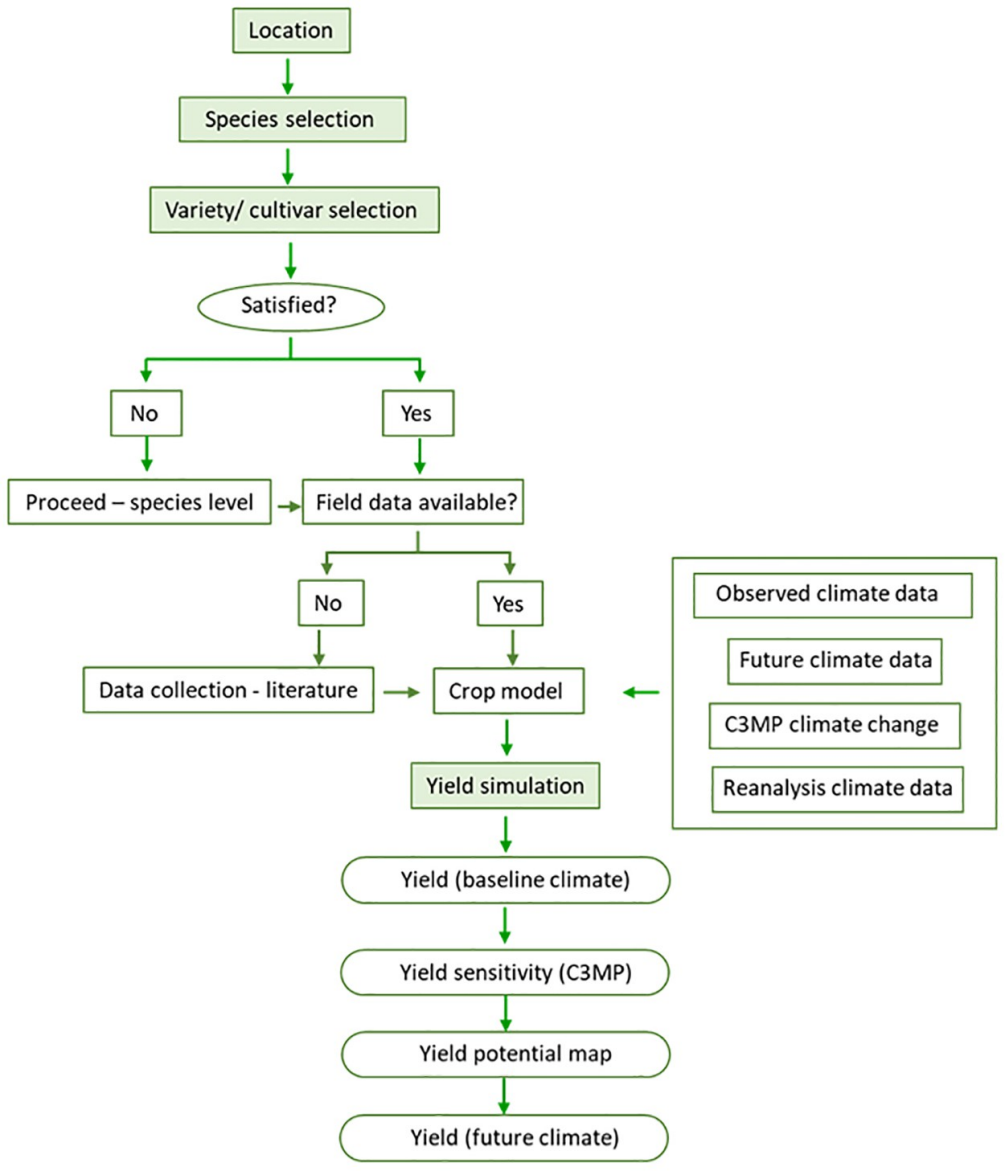
The process flow chart used to study the climate sensitivity of Proso millet.

Yield simulation for other locations and interpolations revealed the potential production of the crop in other locations in Sri Lanka. This is the first study that estimates the potential production for any underutilised crop in Sri Lanka. The findings of the current study can be used as a baseline for detailed field studies on Proso millet in other locations of the country and other underutilised crops globally. Also, a similar approach can be used to assess the potential production of many neglected and underutilised crops in different locations beyond the current niches. Fig 6 presents such a roadmap for the sensitivity analysis of underutlised crops in order to understand the response of different crops to climate change. Such information is invaluable for developing cropping options for the areas that are already affected by climate change and where it is not clear which crop, variety or accession could be suitable. This should be completed with detailed land and climate suitability analysis and detailed economic analysis.

## Conclusions

This paper describes the first study that estimates the climate sensitivity and potential production of Proso millet using a crop modelling approach. The C3MP results revealed that the Proso millet yield increased with up to 2°C of warming at wetter conditions and then decrease with additional warming. In Proso millet growing area, a 1°C increment of temperature decreased the yield by 5–10% at zero rainfall change. The projections for the future climate using 20 GCMs under RCP4.5 and 8.5 scenarios showed a clear increment of annual and seasonal temperatures in the mid-21^st^ century. The studied models showed a possibility of a wetter future in Proso millet growing area for RCP4.5 (85% GCMs) and RCP8.5 (75% GCMs). Proso millet yields of all the tested accessions (L_1, L_11, L_12, L_14 and L_25) were not significantly (p > 0.05) deviated from the baseline yields under both emission scenarios. Potential areas for Proso millet cultivation were identified under both baseline (1980–2009) and future climates. Proso millet yield under climate change showed that yields behave in different ways in different climatic zones. Proso millet, that is mostly cultivated in low input agricultural systems without irrigation and fertiliser, shows both increments and low reductions of yield in the mid-21^st^ century compared to the baseline, suggesting the ongoing suitability of the crop under a changing climate. The yields of key crops like paddy are predicted to fall for similar changes in climate, therefore, Proso millet that is grown with low inputs will be an ideal candidate for marginal areas to provide considerable yield under changing climate in mid-21^st^ century. The framework developed in this study can be used as a baseline study to evaluate the agroclimatic sensitivity of other underutilised crops.

## Supporting information

S1 Fig Distribution of location used in yield mapping (the boundary was obtained from https://data.humdata.org/dataset/cod-ab-lka). (DOCX)

S2 Fig The heatmap for the variation of Proso millet yield change in five different accessions (L_1, L_11, L_12, L_14 and L_25) for all the GCMs individually (A–T in Table 5.3) and the average (Av) of the GCMs for (a) RCP4.5 and (b) RCP8.5. (DOCX)

S1 Table Description of general circulation models (GCMs) used in the study. (DOCX)
