# Supplementary figures and images for: Agro-climatic sensitivity analysis for sustainable crop diversification; the case of Proso millet (Panicum miliaceum L.)
Source: PLoS One. 2023 Mar 23;18(3):e0283298. doi: 10.1371/journal.pone.0283298 (PMC10035905; doi:10.1371/journal.pone.0283298)

**
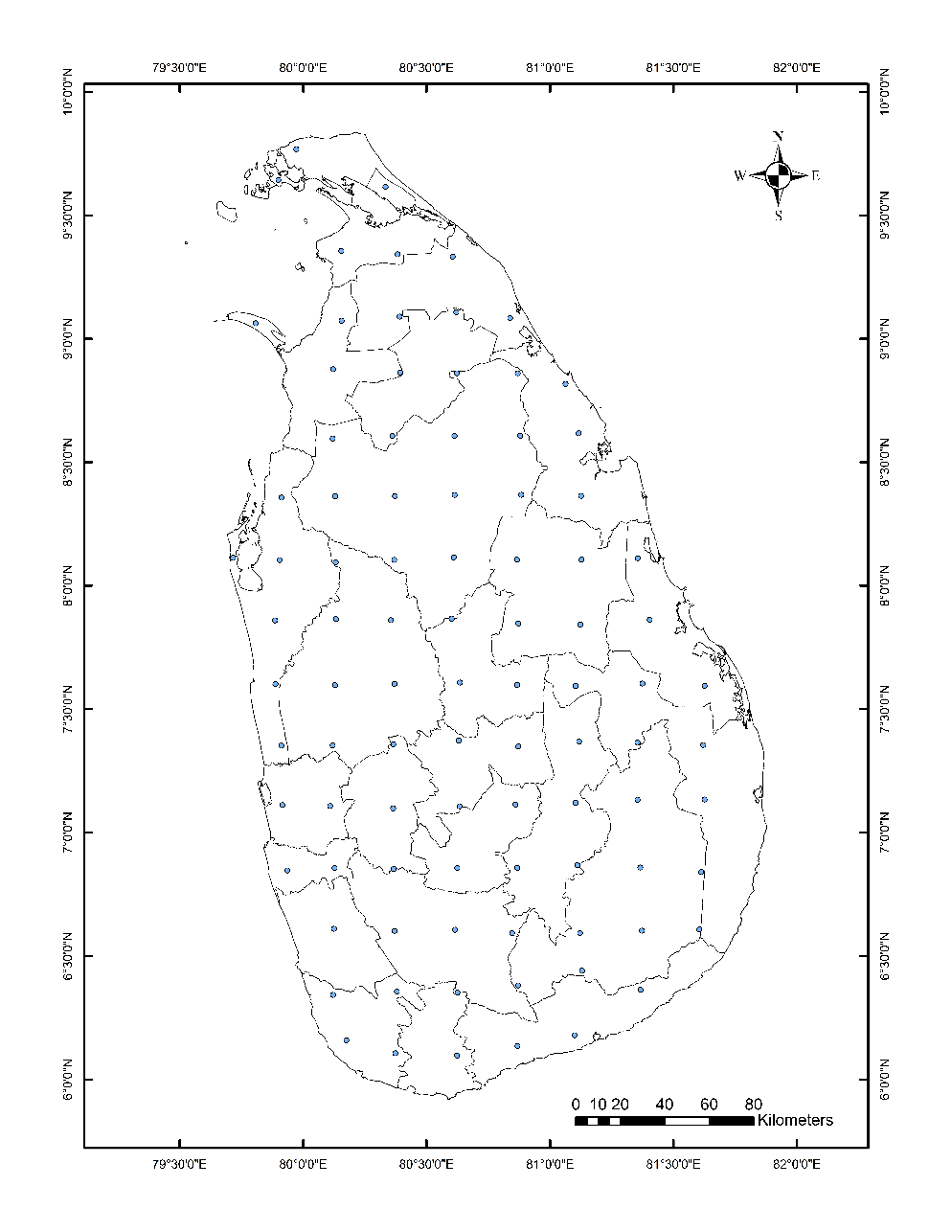
**

**S1 Fig**

Supplement: S1 Fig — (DOCX) [file pone.0283298.s001.docx]

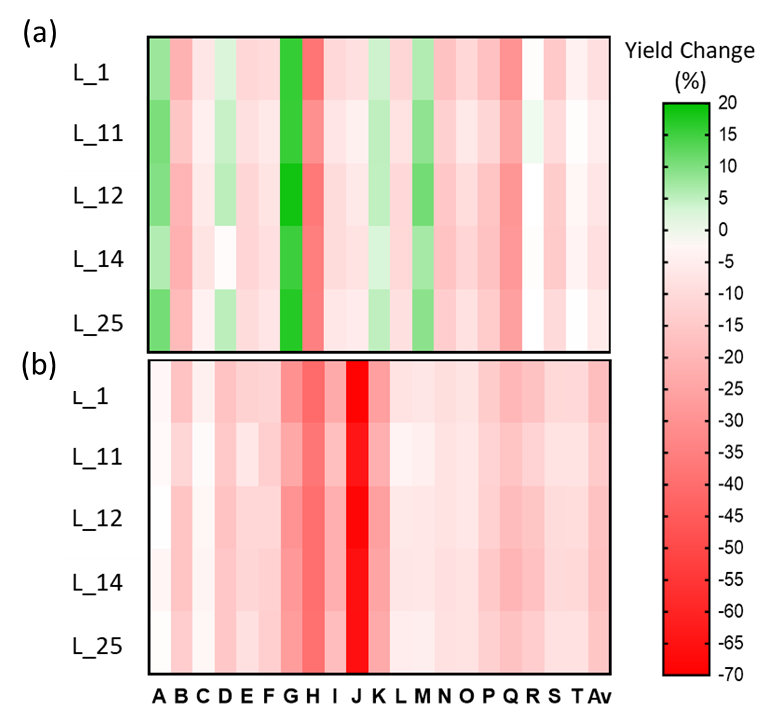


**S2 Fig**

Supplement: S2 Fig — (DOCX) [file pone.0283298.s002.docx]
