# Supplementary material for: Agro-climatic sensitivity analysis for sustainable crop diversification; the case of Proso millet (Panicum miliaceum L.)
Source: PLoS One. 2023 Mar 23;18(3):e0283298. doi: 10.1371/journal.pone.0283298 (PMC10035905; doi:10.1371/journal.pone.0283298)
Supplement: S1 Table — (DOCX) [file pone.0283298.s003.docx]

**S1 Table**

| **Symbol** | **Model** | **Description/Institute** | **Resolution of Atmospheric Grid (decimal degrees)** |
| --- | --- | --- | --- |
| **A** | BCC-CSM1-1 | Beijing Climate Centre, China Meteorological Administration, China | 2.7906 x 2.8125 |
| **B** | BCC_CSM1_1_M | Beijing Climate Centre, China Meteorological Administration | 2.7906 x 2.8125 |
| **C** | BNU-ESM | College of Global Change and Earth System Science, Beijing Normal University, China | 2.7906 x 2.8125 |
| **D** | CanESM2 | Canadian Centre for Climate Modelling and Analysis, Canada | 2.7906 x 2.8125 |
| **E** | CSIRO-Mk3-6-0 | Commonwealth Scientific and Industrial Research Organisation and the Queensland Climate Change Centre of Excellence, Australia | 1.8653 x 1.875 |
| **F** | GFDL_CM3 | Geophysical Fluid Dynamics Laboratory, USA | 2.0 x 2.5 |
| **G** | GFDL-ESM2G | Geophysical Fluid Dynamics Laboratory, USA | 2.0225 x 2.0 |
| **H** | GFDL-ESM2M | Geophysical Fluid Dynamics Laboratory, USA | 2.0225 x 2.0 |
| **I** | INMCM4.0 | Institute for Numerical Mathematics, Russia | 1.5 x 2.0 |
| **J** | IPSL-CM5A-LR | Institut Pierre-Simon Laplace, France | 1.8947 x 3.75 |
| **K** | IPSL-CM5A-MR | Institut Pierre-Simon Laplace, France | 1.2676 x 2.5 |
| **L** | MIROC-ESM | Japan Agency for Marine-Earth Science and Technology, Atmosphere and Ocean Research Institute, and National Institute for Environmental Studies, Japan | 2.7906 x 2.8125 |
| **M** | MIROC_ESM_CHEM | Japan Agency for Marine-Earth Science and Technology, Atmosphere and Ocean Research Institute, and National Institute for Environmental Studies, Japan | 2.7906 x 2.8125 |
| **N** | MIROC5 | Atmosphere and Ocean Research Institute, National Institute for Environmental Studies and Japan Agency for Marine-Earth Science and Technology | 1.4008 x 1.40625 |
| **O** | HadGEM2-CC | Met Office Hadley Centre, UK | 1.25 x 1.875 |
| **P** | HadGEM2-ES | Met Office Hadley Centre, UK | 1.25 x 1.875 |
| **Q** | MPI-ESM-LR | Max Planck Institute for Meteorology, Germany | 1.8653 x 1.875 |
| **R** | MPI-ESM-MR | Max Planck Institute for Meteorology, Germany | 1.8653 x 1.875 |
| **S** | MRI-CGCM3 | Meteorological Research Institute, Japan | 1.12148 x 1.125 |
| **T** | NorESM1-M | Norwegian Climate Centre, Norway | 0.5 |
